# Robot-Assisted Resection of Left Ventricular Papillary Fibroelastoma Arising From the Mitral Chordal Apparatus

**DOI:** 10.1177/15569845231152891

**Published:** 2023-02-13

**Authors:** Kyle G. Mitchell, Blaz Podgorsek, Diego E. Fiorito, Juan A. Abreu, Danny Ramzy

**Affiliations:** 1 Department of Cardiothoracic and Vascular Surgery, University of Texas McGovern Medical School, Houston, TX, USA; 2 Department of Pathology, Memorial Hermann Memorial City Medical Center, Houston, TX, USA

**Keywords:** papillary fibroelastoma, robotic cardiac surgery, minimally invasive cardiac surgery

## Abstract

The application of robot-assisted thoracoscopy to cardiac surgery affords an opportunity to leverage the exceptional intraoperative exposure, visualization, and dexterity of the robotic platform. Here, we report the case of a 72-year-old woman who presented to our institution for evaluation of a left ventricular mass that was identified following workup for an embolic event. We present an intraoperative video that provides technical details of the robot-assisted resection of the lesion, which was found to be a left ventricular papillary fibroelastoma arising from the mitral chordal apparatus. This case highlights the advantages provided by the robotic platform, which permitted complete, minimally invasive surgical excision of the lesion while minimizing the burden of surgical trauma.

## Graphical abstract

**Visual abstract:**
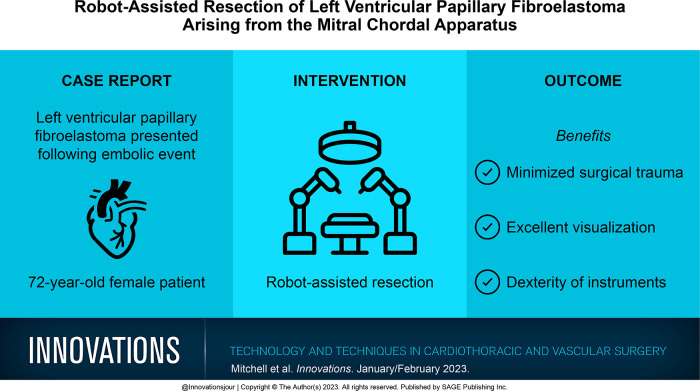

## Introduction

Papillary fibroelastomas are benign proliferative lesions arising from the endocardial surface, most commonly in the left-sided chambers.^1^ Although resection of these lesions has traditionally been achieved via median sternotomy, minimally invasive approaches offer the opportunity to achieve complete excision while minimizing the burden of surgical trauma.^2^ We describe a robot-assisted approach for resection of a left ventricular papillary fibroelastoma arising from the mitral chordal apparatus and provide an intraoperative video demonstrating technical details of the operation.

## Case Report

### Patient Presentation

**Fig. 1.**
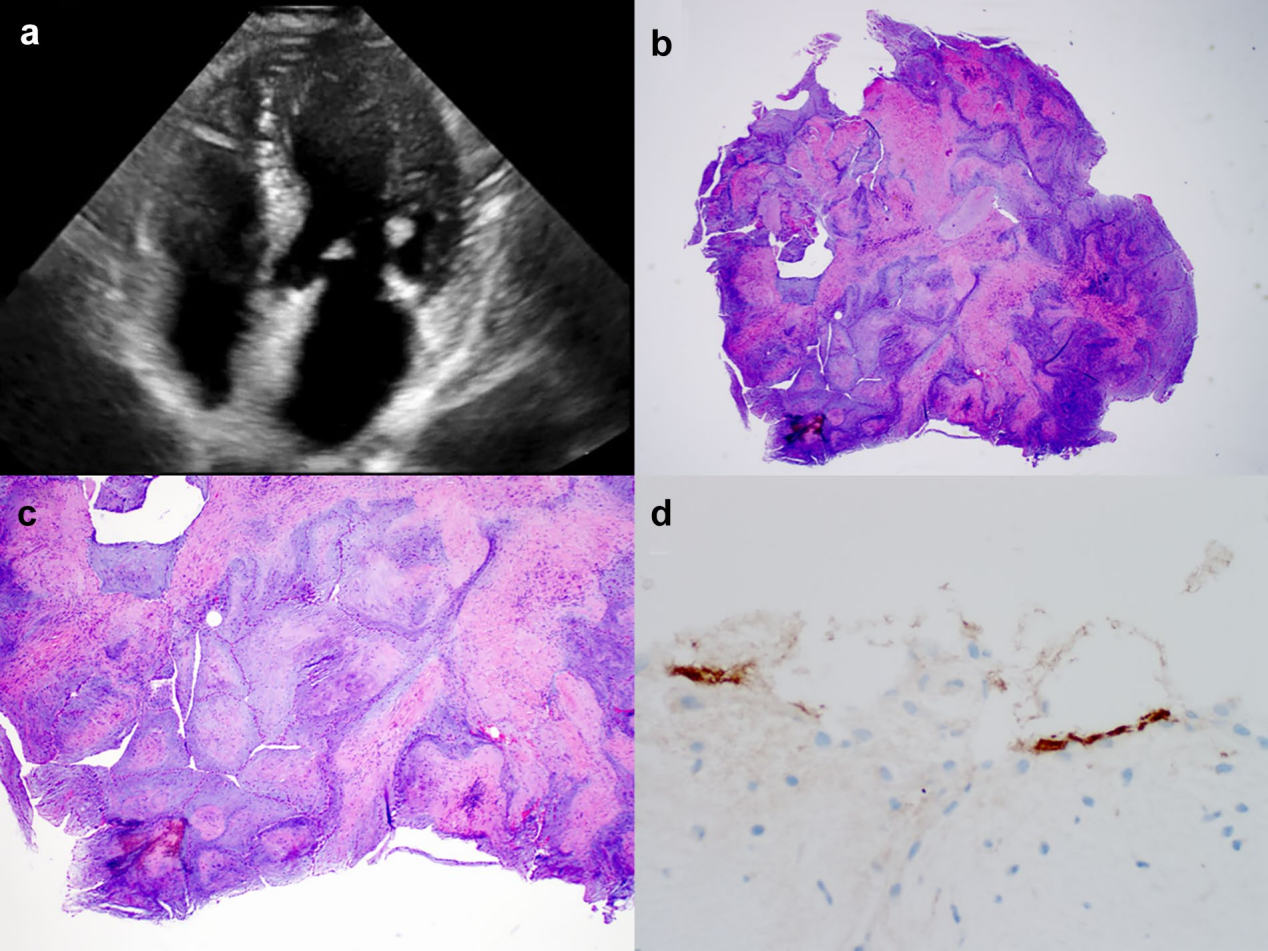
(a) Representative still image from preoperative transesophageal echocardiogram demonstrating a mobile, 8 mm hyperechoic mass in the left ventricle. (b) Low-power view of the resected tumor. (c) Low-power view of blunted avascular cores. (d) Factor VIII immunostain demonstrating endothelial cells lining avascular cores.

The patient was a 72-year-old woman who presented as a transfer to our institution following central retinal artery occlusion with acute unilateral vision loss. Her medical history was notable for hypertension, hyperlipidemia, and tobacco use disorder (current smoker with 52-pack-year history). Diagnostic workup at the outside facility included a transesophageal echocardiogram that demonstrated a left ventricular ejection fraction of 55% to 60%, an 8 mm mobile mass in the left ventricle (Fig. 1a), and a competent mitral valve with no evidence of regurgitation or stenosis. Resection of the intracardiac mass was indicated given the risks of a recurrent embolic event. The present report was exempt from Institutional Review Board review. The patient provided informed consent.

### Operative Details

After induction of general anesthetic and placement of a double-lumen endotracheal tube, the patient was positioned in a similar fashion as for a robot-assisted mitral valve repair, with the right side slightly elevated. The right common femoral artery and vein were dissected and prepared for peripheral cannulation (arterial 17 Fr Bio-Medicus cannula, Medtronic, Dublin, Ireland; venous 22 Fr RAP cannula, LivaNova, London, UK), and systemic heparin was administered.

A limited anterior right thoracotomy was made with a 5 cm incision in the fourth intercostal space above the nipple-areolar complex without rib spreading (Supplemental Video). Additional 8 mm trocar sites were placed 2 intercostal spaces superior (working port), 2 intercostal spaces inferior (working port), and just anterior and inferior (left atrial retractor) to the minithoracotomy. After creation of a pericardial well, placement of an aortic root vent, docking the robot (da Vinci Xi, Intuitive Surgical, Sunnyvale, CA, USA), and initiation of cardiopulmonary bypass, the aorta was cross-clamped via a right lateral chest wall incision. The left atrium was approached via Sondergaard’s groove. A robotic atrial retractor was used to expose the mitral valve. The mass was easily identified in the left ventricle and was found to be arising from the mitral chordal apparatus. The mass was resected in its entirety with limited en bloc resection of its insertion on the chord. The valve was tested and was found to be competent with no regurgitation. The left atriotomy was closed, the heart de-aired, and separation from cardiopulmonary bypass achieved. Postoperative echocardiography demonstrated preserved left ventricular ejection fraction without any evidence of mitral regurgitation. Cardiopulmonary bypass and aortic cross-clamp times were 106 and 52 min, respectively.

The patient’s postoperative recovery was uneventful, and she was discharged home in good condition on the fourth postoperative day. Pathologic examination of the resected mass was consistent with a papillary fibroelastoma (Fig. 1b–d).

## Discussion

Papillary fibroelastomas are pedunculated, frond-like lesions rising from the endocardial surface. They usually arise from a left-sided location and, when associated with a valve (aortic more commonly than mitral), generally arise directly from the valvular surface.^1^ Histopathologically, they are characterized by a matrix consisting of a variable distribution of subendocardial elastin fibers. Clinically, they may be identified upon echocardiographic workup for an embolic event but may also be identified incidentally in an asymptomatic patient or upon autopsy following sudden death.^1,3^ In cases in which echocardiographic findings are indeterminate, cardiac magnetic resonance imaging has been reported to be helpful in distinguishing intracardiac tumor from thrombus.^3^ Given the risk of thromboembolism, particularly for mobile lesions, surgical resection is recommended in medically operable patients.^1^ Excision is curative and recurrence is rare.^4^

Although other groups^5,6^ have previously reported successful less invasive and/or thoracoscopic-assisted excision of papillary fibroelastomas and there is a single previous report of robot-assisted resection,^7^ these lesions have traditionally been approached via median sternotomy.^2,4^ The present report and its accompanying video highlight the utility of the robotic platform to facilitate minimally invasive approaches to lesions arising from the mitral subvalvular apparatus while maintaining excellent exposure, visualization, and dexterity in the surgical field.

In summary, the case and intraoperative video presented here of a resection of a papillary fibroelastoma arising from an uncommon location highlight the technical advantages of a surgical approach leveraging the robotic platform. In light of the benefits afforded by the robotic platform, we feel that minimally invasive approaches to resection of these uncommon lesions should be considered whenever surgeon and institutional experience allow.
